# Sectorial Water Use Trends in the Urbanizing Pearl River Delta, China

**DOI:** 10.1371/journal.pone.0115039

**Published:** 2015-02-25

**Authors:** Mingtian Yao, Saskia E. Werners, Ronald W. A. Hutjes, Pavel Kabat, Heqing Huang

**Affiliations:** 1 Earth System Science, Wageningen University, Wageningen, the Netherlands; 2 Alterra, Wageningen University and Research Centre, Wageningen, the Netherlands; 3 International Institute for Applied Systems Analysis, Laxenburg, Austria; 4 Key Lab of Water Cycle and Related Land Surface Processes, Institute of Geographic Sciences and Natural Resources Research, CAS, Beijing, China; Ecole des Mines d'Alès, FRANCE

## Abstract

Assessing and managing water use is crucial for supporting sustainable river basin management and regional development. The first consistent and comprehensive assessment of sectorial water use in the Pearl River Delta (PRD) is presented by analysing homogenized annual water use data from 2000 to 2010 in relation to socio economic statistics for the same period. An abstraction of water use, using the concept of water use intensity, and based on equations inspired by those used in global water resource models, is developed to explore the driving forces underlying water use changes in domestic, industrial and agricultural sectors. We do this at both the level of the region as a whole, as well as for the nine cities that constitute the PRD separately. We find that, despite strong population and economic growth, the PRD managed to stabilize its absolute water use by significant improvements in industrial water use intensities, and early stabilisation of domestic water use intensities. Results reveal large internal differentiation of sectorial water use among the cities in this region, with industrial water use intensity varying from -80 to +95% and domestic water use intensity by +/- 30% compared to the PRD average. In general, per capita water use is highest in the cities that industrialised first. Yet, all cities except Guangzhou are expected to approach a saturation value of per capita water use much below what is suggested in recent global studies. Therefore, existing global assessments probably have overestimated future domestic water use in developing countries. Although scarce and uncertain input data and model limitations lead to a high level of uncertainty, the presented conceptualization of water use is useful in exploring the underlying driving forces of water use trends.

## Introduction

Global water use has grown at twice the rate of population growth since 1900, amongst others because of urbanization, industrialization and changing life styles [1]. By decreasing water retention capacity and water quality, cities reduce water availability. The expansion of urban areas transforms vegetated covers to sealed concrete surfaces. These changes increase surface runoff [2], alter the impact of precipitation on the water balance, change the fluxes of evapotranspiration and groundwater recharge, and thus affect surface hydrology and reduce water retention capacity in the urban area [3]. In addition, cities discharge massive amounts of pollutants. Especially nutrients and sediments associated with domestic and industrial activities compromise water quality in both surface flows and groundwater [4–7]. At the same time, urbanization alters the temporal and spatial distribution of water uses by changing the population distribution and land use patterns. How to match changing water uses with water availabilities is therefore a key challenge for sustainable water resource management in any heavily urbanized region.

### Assessing anthropogenic water uses

Less effort has generally been put into the assessment of water use than in assessments of water supply [8]. Anthropogenic water use is broadly categorized in three main sectors, i.e. agricultural, industrial, and domestic water uses. For any particular sector, rather data intensive bottom-up water use models have been developed, starting from individual users, in which the sectorial water use is often described as a function of indicators of the production of particular goods or services [9–11]. E.g., in the domestic sector, residential water use is simulated as a function of multiple household characteristics [11–15]. The most common approach for industrial water use analysis estimates the overall industrial water use as a linear function of gross domestic product (GDP) or industrial value added (IVA), depending on the scale analysed, and other influencing factors [16–18]. Water use in thermoelectric power generation plants is mostly estimated as a linear function of actual electricity production [10,19,20]. A brief discussion of the relative definitions of water availability and supply vs water use and demand can be found in section 2.2, a more extensive discussion in S2 Appendix. In this paper we adopt the term “water use”, pragmatically equating it to water supply and avoiding the much less knowable “water demand”.

At the global scale, sectorial water uses are generally approximated as a function of the water used in the production of a unit goods or services [21–23]. Several integrated global water resources assessments that use models based on above mentioned approaches utilized comparable schemes of global water uses. The global LPJmL (Lund-Potsdam-Jena managed Land) model [24] was elaborated by Jachner et al. [25] with withdrawals for households and industry, in addition to water use by global irrigated and rain-fed agriculture. The global water assessment model “WaterGAP 2” [22,26] for instance includes a global water use module capable of assessing current and future water use.

### The objective of this study

On a regional scale, both data intensive sectorial water use models and large scale integrated water use models are of limited use. Data availability from, and consistency between different sources may be better at regional scales than at the global scale because of more uniformity in the statistical methods applied. But details, like household composition or technology selection of different industrial firms, are often not available to support the data intensive assessments. Only few countries conducted sectorial water use surveys that provide detailed and consistent information of water uses on a regional scale [27]. The available data can hardly reveal the development of sectorial water uses and their corresponding driving forces over time, especially in large and fast urbanising regions. Whether or not water is a limiting factor to regional development often remains unknown, as comprehensive overviews of how sectorial water use and its distribution changes are lacking. Therefore, this paper addresses the following questions using the Pearl River Delta (PRD) as a case study:

1. What is the present situation of, and what are the major trends in sectorial water use in the PRD?
2. What socio-economic factors can explain these trends in water use?
3. Can a region of this scale be considered homogeneous in these aspects, or do the cities differ in their development?

To answer these questions, we combine an analysis of homogenized available statistics (section 2.3) with a simple conceptualization of water use and its driving forces that consists of model equations originally developed for global sectorial water uses (section 2.4). Sectorial water uses on the regional and municipal scale can thus be analysed, while driving forces of sectorial water use can also be explored (section 3). Next, study limitations are discussed, followed by a discussion of the implications of our findings for the potential impacts of future socio-economic growth on water requirements (section 4). Finally, conclusions are drawn (section 5).

## Materials and Methods

In this section we describe the studied region, data collection and the proposed conceptualization of water use.

### Study area

**Fig 1:**
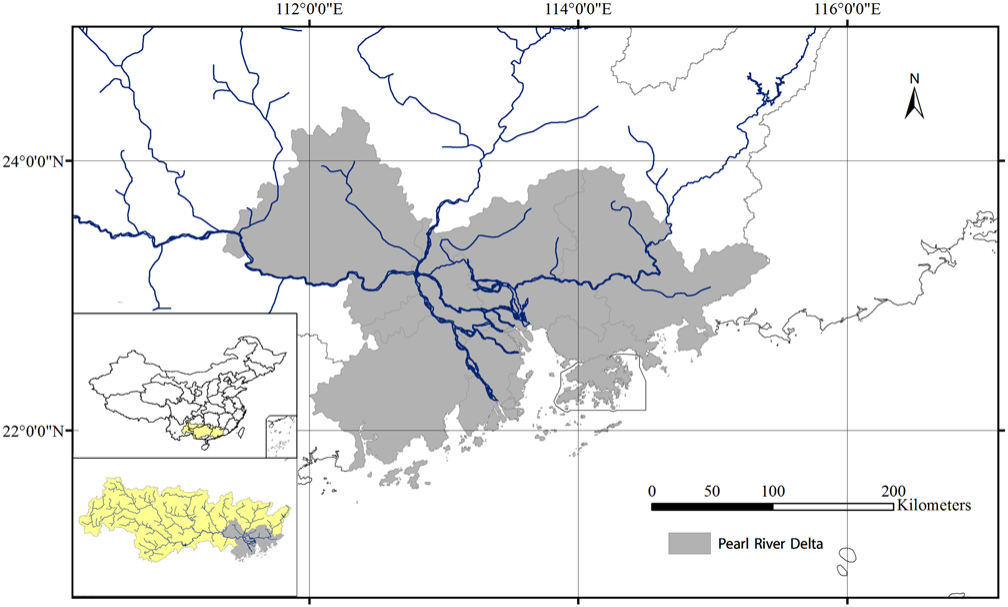
The Pearl River Delta. Map at the lower-left corner is location of the delta area in the whole Pearl River Basin. Above is where the Pearl River Basin is allocated in China.

The PRD, located in Guangdong Province, southeast China (Fig. 1), is the area surrounding the Pearl River estuary that includes nine major urban centres. During the past three decades, it experienced a scale of urban expansion unprecedented in the history of China [28]. As can be observed in many delta areas in the world, recent urbanization development in the PRD area becomes more decentralized, from a single or very few large cities towards a more clustered network of cities that will “dwarf Great London by 26 times” (M. Moore and P. Foster, China to create largest mega city in the world with 42 million people, The Telegraph, 2011) [29,30].

The PRD is among the water abundant regions in China, receiving more than 1700mm average annual precipitation. The Pearl river supplies 95% of the fresh water required in the area [31]. Water from the upstream catchment area converges into a complex distributary system and eventually drains through eight river outlets into the estuary. The discharge of the Pearl River in the delta area varies significantly from less than 4,000 m^3^/s in the dry season to 28,000 m^3^/s in the monsoon season. During a flood event the peak discharge can exceed 40,000 m^3^/s.

Nevertheless, water shortage events have been reported more frequently during the last decade [32–34]. Recent studies indicate that the future water use of the PRD is expected to increase significantly [35,36], while at the same time the Pearl River basin will likely become drier [37,38]. Water shortage appears to become a serious problem for this water abundant delta area [39,40].

### System Boundary

In order to improve our understanding of the driving forces of the water use trends in the PRD, we reviewed the water resource system in the PRD within the following system boundaries.

**Fig 2:**
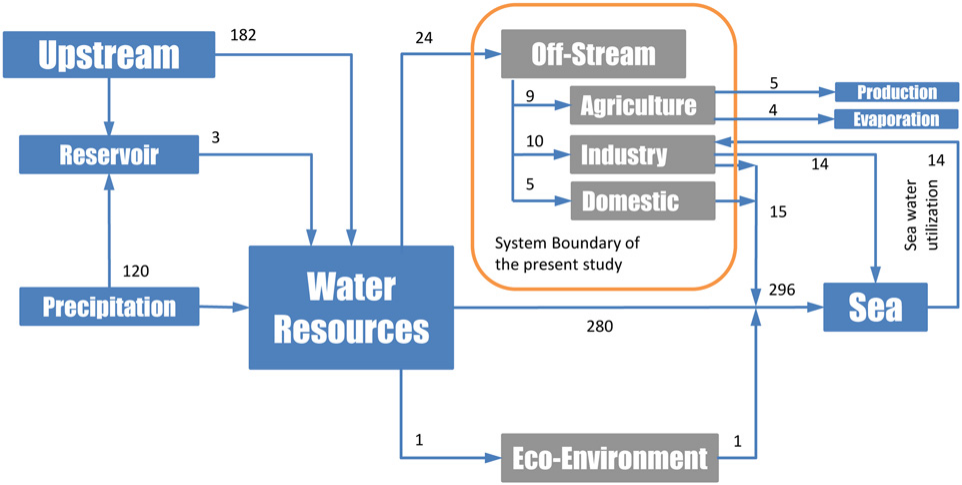
Water resource system of the PRD and the study boundary of the present paper. All the volume numbers are in units of km^3^ (data source: Guangdong Water Resource Bulletin).

As shown in Fig. 2, the PRD water system includes different sources (upstream discharge, impoundment, precipitation and groundwater), and distribution flows (off-stream uses, eco-environment requirements, and direct discharge into the sea). Sea water utilization is also considered, as significant amounts of sea water are used in the region as cooling water for electricity production.

The present study focuses on the off-stream uses of the fresh water resource.

### Data

The main sources of data collected for this study are listed in S1 Appendix.

The “Guangdong Water Resource Bulletin” (WB) is the main source for sectorial water use in the PRD since 2000. By using the five data quality indicators (DOIs) of the “Pedigree Matrix”, we feel the selected data set is appropriate for our study, as it is from the appropriate time period and is collected specifically for the area studied [41]. However, the aspect of completeness is somewhat compromised as the sectorial water uses reported in successive bulletins are not directly comparable, mainly due to changes in the class definitions used in the statistics. Thus the present study homogenizes the WB data in order to overcome the completeness issue and to obtain consistent trends of sectorial water uses.

Here a brief discussion of terminology is appropriate. An extensive discussion of the relative definitions of water availability and supply versus water use and demand can be found in S2 Appendix. The statistics report water withdrawn by, and/or supplied to the various sectors. Since no information is available on processing or conveyance losses, we equate these numbers to (actual) ‘water use’. To assess present or future water stress, one would like to know ‘water demand’ the quantity needed for domestic or socio-economic activities; an amount that cannot always be met. Approaches to quantify demand generally are based on assumptions that relate this to a certain level and measure of economic development. However, exactly these assumptions can be questioned and may not hold when transferred from one situation (country/region, development stage) to another. This indeed is one of the main outcomes of the present study (see our results on domestic water use).

Only, a few major references in Chinese are cited in the main text as we deem appropriate. A more detailed explanation of the Chinese literature we used can be found in S3 Appendix.

### Harmonization of Water Bulletin data

The WB includes annual amounts of water withdrawn by selected sectors and per capita or per unit of output water use intensity for each city in the PRD. However, only water uses in agriculture and industry sectors are reported in consistent categories throughout the study period. Statistical categorization of subpopulations of domestic water users were modified several times.

In order to make the data comparable, we homogenized the WB data to yield total annual amounts of sectorial water use and the corresponding water use intensities for each of the nine cities. We first re-defined water use sectors, thereby separating the manufacturing industry (MAN) from the thermal electricity production (ELE), and grouping the multiple domestic classes into urban (DOMU) and rural (DOMR) domestic sectors, now also consistent over time. Water use volume and water use intensity are calculated then based on WB data and corresponding socio-economic data published. Tables 1 and 2 present the homogenized data of sectorial water use and water intensity, respectively, over the period of 2000 to 2010. Detailed homogenisation steps are described in S4 Appendix.

**Table 1:** Harmonized Sectorial Water Use in 10^8^ m^3^ (Original data can be found in Table A(a) in S4 Appendix).

	Total	AGR	MAN	ELE	DOMU	DOMR
2000	212.9	97.1	-	-	29.2	-
2001	224.7	96.6	58.8	30.0	31.5	6.9
2002	236.4	93.3	-	-	34.7	7.5
2003	249.5	91.2	-	-	40.5	6.4
2004	258.3	89.4	85.0	27.8	46.7	5.6
2005	254.7	86.4	78.9	29.6	46.5	4.9
2006	247.0	83.0	79.5	28.3	51.8	5.1
2007	249.7	81.0	78.1	26.8	50.2	4.9
2008	246.4	81.6	73.3	32.5	48.7	4.9
2009	247.5	80.9	68.8	38.2	48.8	5.0
2010	236.1	74.9	70.6	30.6	48.9	5.2

Agriculture (AGR), Manufacturing Industry (MAN), Thermal Electricity Industry (ELE), Urban Domestic (DOMU), and Rural Domestic (DOMR).

**Table 2:** Harmonized Sectorial Water Use Intensity (Original data can be found in Table B(a) in S4 Appendix).

	ITotal (m^3^/person)	IIND (m^3^/10^4^VA)	IDOMU (l/day)	IDOMR (l/day)	IDOM-Total (l/day)
2000	496	289	269	-	-
2001	513	284	278	150	241
2002	535	264	296	169	261
2003	559	222	335	152	288
2004	572	168	374	139	317
2005	560	132	362	129	309
2006	521	106	377	146	329
2007	506	87	349	136	306
2008	480	71	323	133	286
2009	462	70	308	134	275
2010	420	53	288	148	264

### Conceptualization of Water Use in the PRD

A simple conceptualization of off-stream water use and its driving forces is developed for the PRD that consists of equations reported for globally sectorial water uses. These simple equations are fitted to the harmonized data, to identify driving forces of water uses.

The socio-economic data have been documented more consistently and in greater detail for a longer period. The added value of our approach is that we can make use of these better reported socio-economic data to re-evaluate water use data. In addition, we lay a foundation for a model that can project future water use, assess future water use scenarios.

For each of the 9 cities, we assess fresh water use of four sectors, i.e. domestic (urban and rural separated, DOMU and DOMR), manufacturing industry (MAN), thermal electricity industry (ELE), and irrigation (IRR). In general, water use in every sector is expressed as a function of its driving forces and water use intensity. The total water use is expressed as:

$$W_{Total}=\sum W_{i}=\sum \left(I_{i}\times F_{i}\right)$$ (1)

where *W* is volume of water use, *I*_*i*_ is the water use intensity of sector *i*, and *F*_*i*_ is the driving force of that sector. The water use intensity *I*_*i*_ can be subject to economic, structural and technological changes, as described later. The driving forces *F*_*i*_ are published socio-economic data from the annual statistics (See S1 Appendix for data sources).

#### Domestic

We selected the equation from the global water use model WaterGAP2 and fitted it to each of the nine cities. Although the domestic water use can be simulated as a function of multiple variables such as water price, income, residential consumption and various household characteristics that may affect water use [11–15], the data availability in the PRD is not sufficient to feed these data intensive approaches on household levels.

The approach of WaterGAP2 consists of two main concepts for calculating domestic water use intensity. Firstly, the structural change represents the change in water use intensity as result of the changes in the nature of water-using activities, e.g. behavioural changes or changes in the number of water using appliances [26]. It is represented by a sigmoid function:

$$I_{str}=I_{str-\min}+I_{str-\max}\cdot (1-e^{-\gamma \cdot IN^{2}})$$ (2)

A value of 50 l/person-day was used as the minimum requirement (I_str-min_) [42]. The maximal value (I_str-max_) and the curve parameter (γ) are fitted to each city based on the historical data. The saturated structural water use intensity of the PRD is 430 and 180 litre per person-day for urban and rural respectively, where the corresponding γ is 0.005 and 0.041. Since GDP is normally published as the city average without differentiation between urban and rural sectors, per capita income (IN) of urban and rural resident are used instead in order to separate the urban and rural water use respectively.

The second concept, technological change, is assumed to always improve the efficiency of water use, i.e. decreases the water use intensity. The net domestic water use intensity can be computed by combining structural change and technological change as:

$$I_{DOM}=I_{str}\times {\left(1-\eta \right)}^{t-t_{0}}$$ (3)

where I_DOM_ is the net domestic water use intensity, ŋ is the rate of technological improvements in water use efficiency. A 2% annual improvement rate based on German references from 1950 to 1995 is borrowed from the previous global assessment, as the GDP growth of the PRD during the study period is comparable with Germany in 1970s [26].

The overall domestic water use (W_DOM_) is then expressed as a product of per capita water use intensity (I_DOM_) and population (POP) in the area:

$$W_{DOM}=I_{DOM}\times POP$$ (4)

Urban and rural water uses are separated into two sub-sectors since the observed state and trends of the two strongly differed during the studied period. Only the household water use is computed for the rural domestic sector (DOMR). For urban domestic (DOMU) the public water uses (construction and service industry) are included.

#### Manufacturing Industry

The WaterGAP2 approach for industrial sector is not appropriate for the present study, as it includes fresh water withdrawn for electricity production in the overall industrial water use [26]. In the PRD, the manufacturing sector is comparable with the thermal electricity industry, accounting for about 25% of the total water use. Therefore, we separate them into two sectors.

It is difficult to develop a water use function that can represents all different manufacturing industries accurately in detail, even on smaller scales. Because different cities have different industrial structures (S5 Appendix), and the water use differs between various industries both quantitatively and qualitatively [16–18].

We adopt a simple approach based on a previous study of industrial water use in China where manufacturing water use is computed as a product of the manufacturing water use intensity (I_MAN_) and industrial value-added (IVA) [18]. The manufacturing water use intensity is expressed as a function of per capita GDP and manufacture composition:

$$\ln(I_{MAN})=b_{0}+b_{1}\times \ln(GDP_{ca})+b_{2}\times \ln(R_{HL})$$ (5)

where GDP_ca_ is the annual per capita GDP, R_HL_ is the production ratio between heavy and light industry. The b_0_, b_1_ and b_2_ fitted for the PRD is 10.851, -1.733 and 1.194 respectively. The overall manufacturing industrial water use (W_MAN_)_is then expressed as a product of manufacturing water use intensity (I_MAN_) and IVA of the city (Equation 6).

$$W_{MAN}=I_{MAN}\times IVA$$ (6)

#### Thermal Electricity Industry

Water used by the thermal electricity industry is computed by multiplying electricity production with a regional average water use intensity, I_ELE_. The estimated I_ELE_ varied from 60–100 m^3^/MWh during the study period due to restructuring of the sector (see S1 Appendix for data source). An average of 82 m^3^/MWh is used.

We assume all the electricity in the PRD is produced by thermal power plants. Not all cooling systems need fresh water though. The volume of sea water utilized for cooling purposes in the PRD is subtracted from the result. However the PRD-specific data about sea water utilization are being reported only since 2008. After 2008 67% of Guangdong’s total seawater use for cooling occurred in the PRD. The same ratio is also applied to the period before 2008.

#### Irrigation

The consumptive irrigation water use is computed as the amount of water required by crops to be able to transpire at the optimal rate under the given climate conditions. Crop specific consumptive water use intensity I_c_ is computed following the FAO-56 approach based on 10-days intervals [43]. Irrigation water withdrawal is then estimated with a local irrigation efficiency of 0.6 (see S1 Appendix for data source).

Daily meteorological data are gathered from 8 measurement stations in the area. Cultivation areas of 15 crops are collected for each of the nine cities. A PRD-specific paddy rice crop factor and the national average factors for vegetable, cash crops, banana and orange are listed in Tables 3, 4 and 5 (see S1 Appendix for data source). For other crops the FAO suggested global average value are adopted [43]. Vegetable, fruits and alfalfa are assumed to be grown all year around. Crop factors for these plants remain constant through the year.

**Table 3:** Monthly Crop Factors for Paddy Rice in the PRD Area.

	MAR	APR	MAY	JUN	JUL	AUG	SEP	OCT	NOV
Early Rice	1.65	1.47	1.48	1.44	1.31				
Second Rice				1.41	1.16	1.37	1.54	1.53	1.33

**Table 4:** Crop factors for other cereals and cash crops.

	Initial	Develop	Middle	End
Other Cereals	0.30	0.73	1.15	0.40
Tubers	0.50	0.80	1.10	0.95
Soybean-Spring	0.40	0.78	1.15	0.50
Soybean-Summer	0.40	0.78	1.15	0.50
Groundnuts-Spring	0.40	0.78	1.15	0.60
Groundnuts-Autumn	0.40	0.78	1.15	0.60
Sugarcane	0.40	0.83	1.25	0.75

**Table 5:** Crop factors for fruits, vegetables and green fodder.

	K_c_
Banana	0.90
Orange	0.79
Alfalfa	0.95
Vegetables & Melons	0.79
Other Fruits	0.75

## Results

### Sectorial Water Use

**Fig 3:**
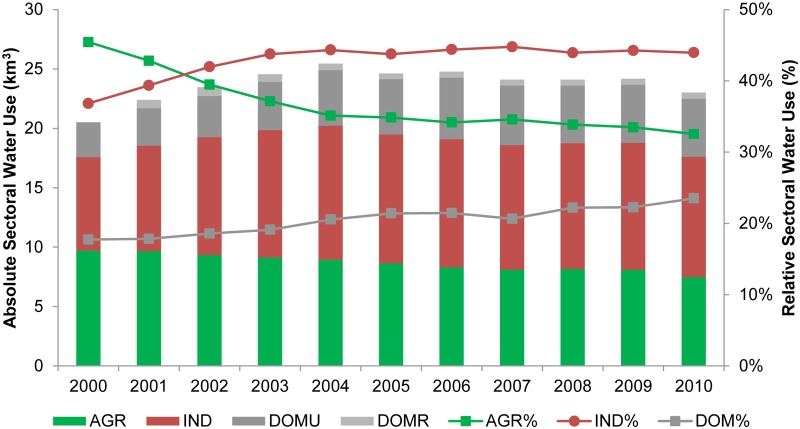
Sectorial water use of the PRD. Absolute (bars, left axis) and relative (lines, right axis) sectorial water use of agriculture, industry and domestic water use of the PRD reported by Guangdong Water Resource Bulletin.

Fig. 3 presents the homogenized sectorial water uses in the PRD during the period 2000 to 2010. On average about 24 km^3^ fresh water was used annually. Industry (comprising manufacturing and electricity generation) surpassed agricultural water use in 2002, accounting for 43% of the total water use and remained rather stable afterward in both percentage and absolute amount. Agriculture water use gradually decreased from 9.7 km^3^ in 2000 to 7.5 km^3^ in 2010, accounting for 45% and 32% of the total respectively. Domestic water use increased by 5% relative to the total, from 3.8 km^3^ in 2000 to 5.4 km^3^ in 2010.

### Sectorial Water Use Intensity

**Fig 4:**
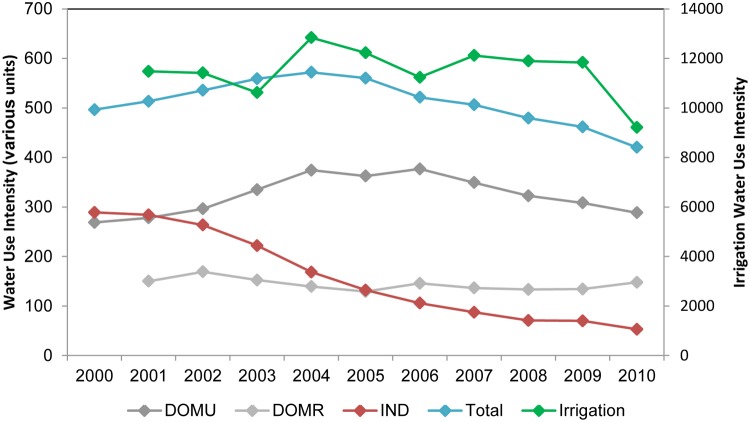
Water use intensity in the PRD. Values shown are in the units of litres/person-day for DOMU and DOMR, m^3^/10^4^ Yuan IVA for IND, m^3^/ha for IRR, and m^3^/person-year for the Total water use intensity (Total) respectively.

As shown in Fig. 4, industrial water use intensity in the PRD is decreasing, while water use intensities remained rather stable in the rural, domestic and irrigation sectors. Urban domestic and the overall per capita water use intensity share a similar trend that peaked in 2004 and gradually decreased thereafter. On average, people consumed 288 litres of water per day for domestic uses. The industry sector needed on average about 160 m^3^ of water to produce 10,000 Yuan of IVA. Crops required 11,500 m^3^ per ha for irrigation. In total an average of about 500 m^3^ of water was used per capita in the PRD during the studied period.

### Exploration of Driving Forces

The conceptualization of water use was developed to get better insights and understanding of the underlying driving forces of water use development in the PRD. It allows us to use better documented socio-economic data evaluate water uses during the studied period.

**Fig 5:**
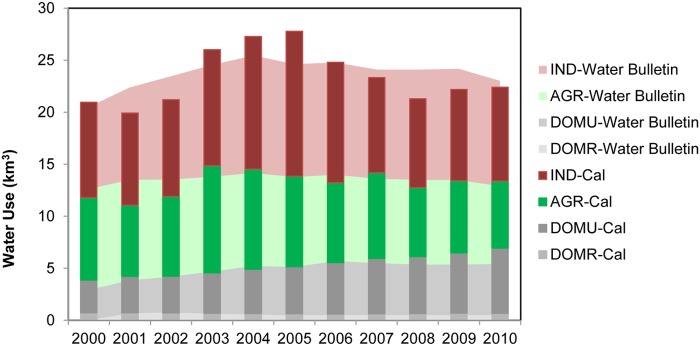
Sectorial water use comparison between calculated results and WB reported data.

**Fig 6:**
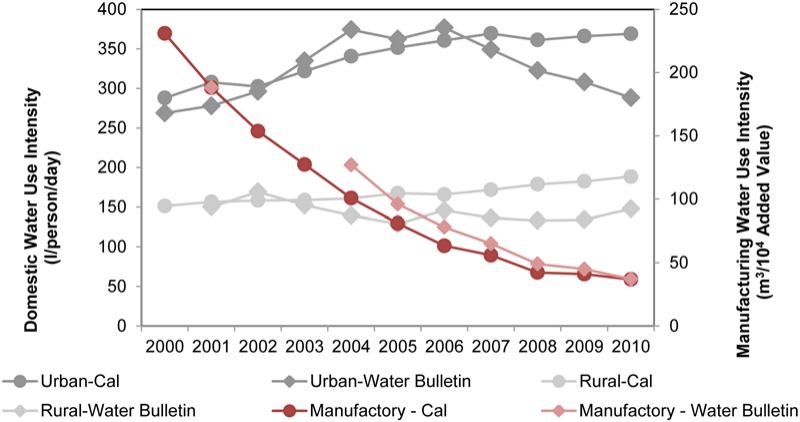
Comparison of domestic and manufacturing water use intensity between calculated results and WB reported data.

Results of absolute volume and intensity of the sectorial water uses in comparison with the homogenized WB records are presented in Figs. 5 and 6 respectively. The conceptual framework with globally reported sectorial water use equations explains well the sectorial water uses with the selected socio-economic variables as listed in Table 6 for the studied period.

**Table 6:** Socio-economic Development in the PRD.

	Population 10^4^ person	Urban Population 10^4^ person	Rural Population 10^4^ person	IVA 10^8^ Yuan	R_HL_	GDP per capita Yuan	Cultivation Area ha
2000	4,290	2,981	1,309	2,724	98%	18,815	732,701
2001	4,376	3,109	1,268	3,128	104%	20,295	719,500
2002	4,415	3,205	1,210	3,763	105%	22,657	688,618
2003	4,464	3,312	1,151	4,841	114%	26,292	657,420
2004	4,517	3,421	1,095	6,695	114%	30,866	761,667
2005	4,547	3,516	1,031	8,217	126%	36,118	634,379
2006	4,737	3,769	969	10,188	131%	41,774	559,291
2007	4,931	3,937	994	12,020	128%	47,892	622,056
2008	5,138	4,134	1,004	14,954	131%	53,310	687,015
2009	5,362	4,337	1,025	15,286	128%	54,789	598,254
2010	5,616	4,646	970	19,080	129%	61,757	524,247

The levelling off trend of the urban domestic water use intensity was well reproduced by income. The calculated rural water use intensity increased gradually following the income, but a rather stable trend was recorded.

**Fig 7:**
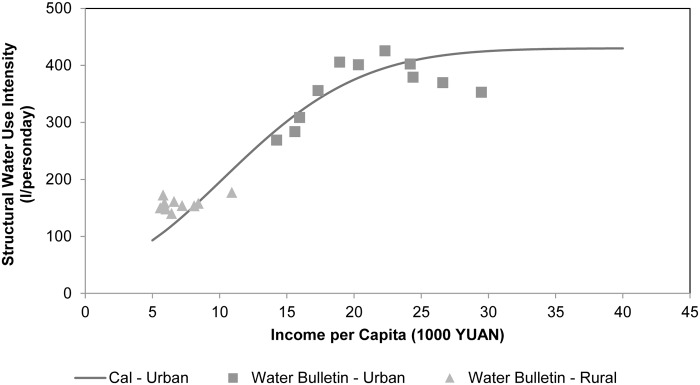
Structural water use intensity in the domestic sector in the PRD. Comparison between calculated results and WB reported data.

Domestic water use intensity in the PRD shows a similar trend as in the global assessment, as shown in Fig. 7 [26]. Results for the better developed urban sectors correspond to the levelling off or stable part of the curve, suggesting the urban domestic sector has reached its saturation water use intensity, and should remain stable or even start to decrease as is suggested by trends observed in global assessments. But we failed to reproduce the significant decline after 2006. Results for the rural domestic sector correspond to the steep part of the curve, which implies that in rural areas water use intensity may still grow sharply with income increases.

The presented conceptualization also explains well manufacturing water use intensity, as shown in Fig. 6, with per capita GDP growth, manufacture composition, and IVA.

### Internal Differentiation

**Fig 8:**
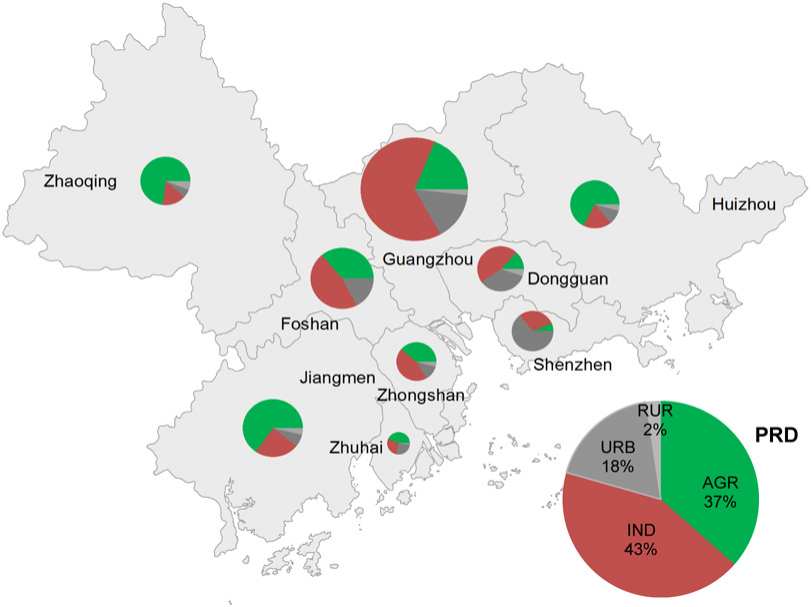
Sectorial water use of different cities in the PRD. Size of the pie chart indicates the average total water use level of the city over the study period. Areas of green, red, dark grey and light grey represent the proportion of agriculture, industry, urban domestic and rural domestic water uses respectively.

Fig. 8 illustrates the relative size of sectorial water use in the different cities and the whole PRD. The nine cities show substantial differences. Guangzhou, which is the capital of the area and inhabits more than 20% of the population of the PRD, accounted for one third of the total water use. It was the dominant water user for industry and urban and rural domestic sectors, accounting for 51%, 29% and 21% of the total on average respectively. Jiangmen was the largest agricultural water user accounting for 22% of total agricultural water use.

Water use intensity show significant differences as well between cities, especially in the manufacturing and domestic sectors. In general, domestic water use intensity was higher in the cities that industrialised early. Guangzhou citizens consumed the most among the nice cities with 386 litres of water per person-day, whereas people in Zhaoqing required 226 litres only. Since 2008 Zhuhai has surpassed Guangzhou requiring the most water for per capita urban domestic use.

The nine cities also had varying saturation water use intensity during the studied period as can be seen in Table 7. Guangzhou required the most at more than 530 litres per person-day, which was comparable to the maximum recorded for the United States and European countries [44,45]. Zhaoqing however had much lower value of 300 litres per person-day only.

**Table 7:** City differentiation of domestic water use parameters.

City	γ_UR_	I_str-max-UR_ l/person-day	R^2^_UR_	γ_RU_	I_str-max-RU_ l/person-day	R^2^_RU_
Dongguan	0.003	440	0.847	0.008	260	0.866
Foshan	0.006	330	0.761			
Guangzhou	0.005	530	0.392	0.017	290	0.228
Huizhou	0.007	310	0.870	0.084	130	0.583
Jiangmen	0.008	370	0.758	0.033	140	0.749
Shenzhen	0.002	430	0.865	0.015	150	0.995
Zhuhai	0.005	400	0.841	0.043	130	0.569
Zhaoqing	0.011	300	0.956	0.100	120	0.403
Zhongshan	0.005	380	0.670	0.020	210	0.073
PRD average	0.005	430	0.855	0.041	180	0.601

Before 2003 Guangzhou required most water per person-day in the rural domestic sector. Since then it dropped significantly. Rural domestic water use intensity showed a downward trend in most cities. Dongguan, however, showed a significant increase from 83 litres per person-day in 2000 to 254 litres in 2010. No rural domestic water use occurred in Foshan and Shenzhen since 2002 as the two cities are fully urbanized.

Looking at the water use for irrigation, Zhuhai, Dongguan and Shenzhen show remarkably lower intensities than the PRD average. Irrigation water use per ha in Dongguan was less than 6,700 m^3^, followed by Zhuhai and Shenzhen with less than 8,000 m^3^. Irrigation systems in the other cities required more than 10,000 m^3^/ha of water. The average irrigation water use intensity in the PRD during the studied period was 11,490 m^3^/ha.

With respect to industrial water use required to produce 10,000 Yuan IVA, the nine cities showed great diversity in 2000. The manufacturing industry in Zhaoqing required 1,073 m^3^, which is 25 times the 42 m^3^ for Shenzhen. All the cities exhibited remarkable improvements of the manufacturing water use intensity afterwards. By 2010, the average amount of water used in the manufacturing industry to produce 10,000 Yuan IVA was 58 m^3^ in the PRD. Shenzhen had the most water effective manufacturing industry requiring 12 m^3^ only, whereas Guangzhou showed the highest value of 114 m^3^. Table 8 shows the curve parameters (*b*_*0*_, *b*_*1*_, *b*_*2*_) fitted for each of the nine cities based on historical water uses. As can be seen, the manufacturing structure (*b*_*2*_) had significant influence on water use intensity in the earlier industrialized cities such as Guangzhou and Zhaoqing, whereas a newly established city like Shenzhen was mostly affected by the economic development (*b*_*1*_).

**Table 8:** Parameter fitting for the manufacturing water use calculation.

City	b_0_	b_1_	b_2_	R^2^
Dongguan	7.957	-0.971	-0.480	0.953
Foshan	9.313	-1.419	0.284	0.950
Guangzhou	17.865	-3.363	2.326	0.991
Huizhou	3.363	0.356	-0.435	0.572
Jiangmen	10.885	-1.826	1.562	0.903
Shenzhen	7.803	-1.130	-0.033	0.944
Zhuhai	6.410	-0.677	-0.497	0.933
Zhaoqing	10.021	-2.107	1.755	0.801
Zhongshan	7.472	-0.787	-0.002	0.968
PRD average	10.851	-1.733	1.194	0.992

**Fig 9:**
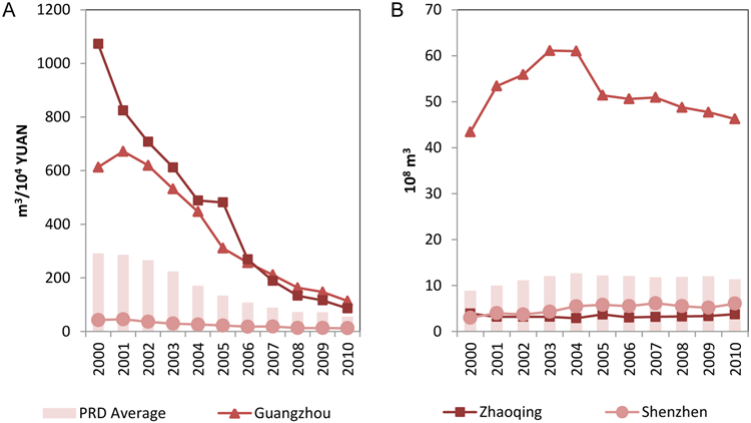
Internal differentiation of water use in the manufacturing sector in the PRD. Fig. 9A is the internal differentiation of the manufacturing water use intensity. Fig. 9B is the internal differentiation of absolute volume of manufacturing water use.

Fig. 9(a) shows the highest and the lowest manufacturing water use intensity at city level and their development in comparison with the PRD average, whereas Fig. 9(b) presents the absolute volumes of water used. Regardless of the significant improvement of the intensity, the absolute volume of manufacturing water use remained rather stable for all cities, due the fast economic growth.

## Discussion

In this study we present the first consistent and comprehensive assessment of water use in all cities of the PRD. Previous local studies estimated sectorial water use by assuming typical per-capita intensities that were either depend on the size of the city [46], or a single average value as the baseline with fixed growth rate [47]. In our conceptual framework, city-specific water use data and corresponding socio-economic figures are linked by selected globally reported model equations.

### Uncertainties from water use data

The water use dataset collected from the Water Bulletin is in general well documented. By checking with the Pedigree Matrix, data quality regards to reliability and geographical correlation are both very high. Due to the compromised completeness, we performed harmonization in order to mitigate the uncertainties.

As a consequence of the change in reporting of census data, the urban per capita water use intensity may be overestimated, while the rural figure may be underestimated. The “HuKou” registration system in China, which manages people in their birthplace rather than place of residence, results in underestimation of urban populations by ignoring migration labourers. It is also unknown whether the absolute volume of water use by this “ignored” population was taken into account, as it was not specified in the statistics before 2003.

Lack of data also caused uncertainties in the calculated results, especially for the industrial sector. The thermal electricity industry is poorly represented. Both absolute volume and intensity of water use in this sector were hard to access, as were the details about the thermal power plants, e.g. type of cooling system, or technology adopted. Calculations based on an average provincial water use intensity quota thus may have overestimated water use, as the better developed delta area may have adopted more efficient cooling system than the provincial average. Elaboration of the water use estimation in electricity production sector will require more details about the configuration of power plants in the PRD and their use of sea water vs fresh water for cooling.

In addition, issues such as illegal withdrawal are not considered in our study because of lack of data. Water use will be underestimated if unregistered rural domestic and industrial withdrawal occurred.

### Uncertainties from socio-economic yearbooks

The published IVA includes only the enterprises above a certain threshold which account for around 83% of the total industrial value added in 2000 to above 90% in 2010. The total amount of manufacturing water use may be underestimated due to the incomplete IVA data. More in general, the regional water use of the industrial sector and the relevant impact factors are still poorly understood due to the complexity of industrial structure, scale, technological development and policy.

### Comparing to previous Global Water Use Assessments

As WaterGAP2 suggests, while income increases, the simulated structural water use intensity levels off, depending on the saturation value selected. Previous global assessment with WaterGAP2 suggested that for developing countries a saturation value comparable with the United States and Europe should be adopted, as these regions are expected to follow the same development trajectory and finally reach a comparable maximum intensity [26]. However, the reported water use intensities for most PRD cities do not support this scenario, and large internal differentiation exists. The average saturation structural water use intensity of the PRD is significantly less than the values used in global assessment as about 425 litres per person-day as shown in Fig. 8. City-specific values varied from 300 to 530 litres where early developed cities like Guangzhou have much higher values than recently developed cities like Zhaoqing.

One probable reason for such differentiation between PRD cities is that households in later developed cities started with more sophisticated water use appliances and technologies and are supported by a better supply system. As reported by Chu et al. [48], water use appliances have different diffusion paths among households along with the income growth. Appliance replacement has its impacts on water use efficiency. This implies the parameter *I*_*str-max*_ in the Equation 2 should not be set to a global constant, but requires location-specific historical records.

Recent elaboration of WaterGAP2 also reported similar differentiation on saturation value for European countries [44]. Our study confirms that differentiation exists not only among countries, but on smaller scales as well. Fast developing delta areas like the PRD are unlikely to reach domestic water use intensities as high as the global assessments suggested. Water requirement scenarios for the domestic sector in developing countries are very likely overestimated in previous global assessments.

In comparison with the most recent global estimation, manufacturing water use intensity in the PRD of about 67 m^3^/1,000 US$2000 IVA was already better than 86 m^3^ of the Asian-Pacific average. Nevertheless, although the water use intensity was reduced by half over the studied period, the level of 30 m^3^ in 2010 was still much higher than the North American value of 19 m^3^ in 2005 [17].

With regards to the electricity generation sector, the 82 m^3^/MWh intensity used for the present study is still higher than the up-to-date once-through system of 76 m^3^/MWh. The most water efficient cooling pond requires only 1.1 m^3^/MWh [10].

### Study Limitations and Outlook

The temporal correlation aspect of data quality is bit compromised as the water use data are available for only ten years, which limited our study period no earlier than 2000s. Ideally our study may provide more comprehensive results if the data could support an analysis for the last 30 years, because the PRD started its economic booming since late 1970s. Also, longer time series might have allowed a separation of parameter fitting and their validation to different subsets, i.e. sub periods of the data, an approach we believed of limited use within the current dataset limitations. Also, the reported strong differentiation between cities did not leave room for validating parameters found for one city to be validated at another.

Reasons for the mismatch in domestic water use intensity between calculated results and reported data after 2006 may be caused by the fact that the domestic water use equation of WaterGAP2 does not include any explicit parameter representing water savings, e.g. due to water pricing and public awareness improvement. Apparently, the rate of technological improvements in water use efficiency (*ŋ* in equation 3) cannot represent such factors as the policy changes induces a stepwise improvement at such small scale, rather than a more gradual improvement as a policy disperses through a large country. Factors such as policy regulation and raising public awareness should be included, as central administrative measurements have strong impacts in China. Public awareness and habits of water saving as the results of recently promulgated laws and regulations significantly improved domestic water use intensities since 2003 [46].

Manufacturing water use intensity differs strongly between the PRD cities due to the diverse industrial structure and historical development. Take Guangzhou and Shenzhen as example: the IVA of these two cities was similar in 2000 at 70 billion Yuan. But the water use intensity of Guangzhou was 15 times larger than that of Shenzhen. Statistics show that heavily water dependent manufacturing industries such as chemical materials, textile and petroleum accounted for more than 40% of gross output value of the Guangzhou industry. While 40% of the gross output of Shenzhen industry was generated by electronic equipment production. The high-tech industry was selected as the economic engine for Shenzhen for the new special economic zone set in 1978, when China launched the reform and opening-up policy, which resulted in a low industrial water use intensity from the very beginning of its development. We suggest further analyses of the impact of different industrial development trajectories on water use in the future.

Both WB water use data and calculated results suggest that on the annual scale water use in the PRD is well managed without considerable shortages. Yet, severe seasonal water shortages have been reported due to temporarily unevenly distributed water resources in the delta area(Q. Chen, Half the Guangdong Municipalities Encountered Water Shortage, Nanfang Daily, 2010). Water shortage caused jointly by drought and salt intrusion requested 843 million m^3^ of fresh water from upstream reservoirs to relieve the stress in 2005, followed by 550 million m^3^ in 2006, which is equivalent to about one sixth of the annual urban domestic water use. Such events indicate that the water resource management system in the delta area is vulnerable to changes in the hydrological regime. Achieving sustainable water management in the PRD will require more insight in seasonal fluctuations in future studies. Furthermore, as water use is only part of the water resource management, to better understand the water scarcity in the PRD from a systematic perspective, a full life cycle analysis (LCA) on water resource could be very helpful. Recent works on cascading effects within a single river basin [49] and environmental impacts from different components of the entire urban water system [50] provide good examples.

## Conclusion

Analysis of homogenized observation data and the presented conceptualization of water use provided the first consistent and comprehensive assessment of sectorial water use across the nine cities in the PRD during the period of 2000–2010. The homogenized sectorial water use volumes and intensities revealed clear trends in water use in the PRD. The conceptualization of water use offers the advantage to explore insights of driving-forces of such trend by linking reported socio-economic data with annual water use data.

On average about 24 km^3^ fresh water was used annually in the PRD. Industrial water use surpassed agriculture and became the dominant water user in 2002, accounting for 43% of the total water use.

Large internal differences exist between cities. In general the early developed cities have higher domestic water use intensity. Per capita water use in all cities except Guangzhou reached a saturation value much below what is suggested in recent global studies. Thus the current global outlooks may overestimate domestic water demand for developing countries.

Water use in the manufacturing sector showed even larger differentiation between cities due to a great diversity of industrial structure, scale, technologies and policy decisions. City like Shenzhen selected high-tech manufactures to be the pillar industry from the very beginning of its industrialization. Consequently, its manufacturing water use intensity is found to be much lower than in other cities.

Despite the fast growth in economy and population, the PRD managed to stabilize its absolute water use by improving water use intensities. Nevertheless temporary shortages occur. To better understand the temporal and spatial distribution of water use and potential shortages, it is important to have more insights on monthly resolution and to catch the complexity of manufacturing sector at the regional level. Finally the impact of water related regulations and improvement of public awareness should be taking into account in order to assess whether fresh water will become a limiting factor for socio-economic development in the PRD.

## Supporting Information

S1 Appendix Data Sources. (DOCX)

S2 Appendix Schematic Definition of Water Supply/Demand/Use. (DOCX)

S3 Appendix Chinese language references. (DOCX)

S4 Appendix Data Harmonization. (DOCX)

S5 Appendix Top 10 Manufacturers in the PRD. (DOCX)
