# Supplementary material for: Sectorial Water Use Trends in the Urbanizing Pearl River Delta, China
Source: PLoS One. 2015 Feb 25;10(2):e0115039. doi: 10.1371/journal.pone.0115039 (PMC4340799; doi:10.1371/journal.pone.0115039)
Supplement: S1 Appendix — (DOCX) [file pone.0115039.s001.docx]

## Appendix S1. Data Sources

| No. | Data Source | Resolution^1^ | Accessed^2^ | Publisher |
| --- | --- | --- | --- | --- |
| 1 | China Energy Statistical Yearbook | Nat. | Nat. Lib. | NBSC^4^ |
| 2 | Pearl River Water Resource Bulletin | Reg. | Online^3^ | PRWRC^5^ |
| 3 | Daily Surface Climate Dataset | Reg. | Online | NMIC-CDC^6^ |
| 4 | Yangtze River Delta & Pearl River Delta and Hong Kong & Macao SAR and Taiwan Statistical Yearbook | Reg. | Nat. Lib. | NBSC |
| 5 | Agricultural Statistical Yearbook of Guangdong | Pro. | Nat. Lib. | Guangdong Agri. Dep. |
| 6 | Guangdong Water Resource Bulletin | Pro. | Online | Guangdong Water Resource Dep. |
| 7 | Guangdong Statistical Yearbook | Pro. | Nat. Lib. | Guangdong Stat. Bur. |
| 8 | Dongguan Statistical Yearbook | Mun. | Nat. /Pro. Lib. | Dongguan Stat. Dep. |
| 9 | Foshan Statistical Yearbook | Mun. | Nat. /Pro. Lib. | Foshan Stat. Dep. |
| 10 | Guangzhou Statistical Yearbook | Mun. | Nat. /Pro. Lib. | Guangzhou Stat. Dep. |
| 11 | Huizhou Statistical Yearbook | Mun. | Nat. /Pro. Lib. | Huizhou Stat. Dep. |
| 12 | Jiangmen Statistical Yearbook | Mun. | Nat. /Pro. Lib. | Jiangmen Stat. Dep. |
| 13 | Shenzhen Statistical Yearbook | Mun. | Nat. /Pro. Lib. | Shenzhen Stat. Dep. |
| 14 | Zhaoqing Statistical Yearbook | Mun. | Nat. /Pro. Lib. | Zhaoqing Stat. Dep. |
| 15 | Zhongshan Statistical Yearbook | Mun. | Nat. /Pro. Lib. | Zhongshan Stat. Dep. |
| 16 | Zhuhai Statistical Yearbook | Mun. | Nat. /Pro. Lib. | Zhuhai Stat. Dep. |
| 17 | Quick-speed calculation of Agriculture Handbook (Vol.2): crop cultivation (including field testing). plant protection. agricultural and water volume | Pro. | Nat. Lib. | Chemical Industry Press |
| 18 | Elementary Discussion on Water Use Efficiency of Irrigation District | Nat. | http://en.cnki.com.cn/ | China Rural Water and Hydropower^7^ |
| 19 | Guangdong Water Use Quota | Pro. | Online^8^ | Guangdong Water Resource Dep. |

1. Data availability on different spatial scales, i.e. national (Nat.), regional (Reg.), provincial (Pro.) and municipal (Mun.)
2. Data accessibility in different sources, i.e. national library (Nat. Lib.), provincial library (Pro. Lib.), and online database
3. Online data can be accessed at:
   - <http://www.pearlwater.gov.cn/xxcx/szygg/>
   - <http://cdc.cma.gov.cn/>
   - <http://www.gdwater.gov.cn/yewuzhuanji/szygl/szygb/>
4. National Bureau of Statistics of China
5. Pearl River Water Resource Commission
6. Climatic Data Center, National Meteorological Information Center of China
7. Scientific journal in Chinese. English abstract is available at <http://en.cnki.com.cn/Article_en/CJFDTOTAL-ZNSD200307009.htm>
8. <http://www.gd.gov.cn/govpub/rdzt/deys/>
