# Supplementary material for: Sectorial Water Use Trends in the Urbanizing Pearl River Delta, China
Source: PLoS One. 2015 Feb 25;10(2):e0115039. doi: 10.1371/journal.pone.0115039 (PMC4340799; doi:10.1371/journal.pone.0115039)
Supplement: S2 Appendix — (DOCX) [file pone.0115039.s002.docx]

## Appendix S2. Schematic Definition of Water Supply/Demand/Use

“Water supply” in this paper refers to all the water provided to end users by either centralized waterworks or self-extraction. The term “water demands” and “water use” are not synonymous, as the “demand” cannot always be fulfilled when water deficit exists.

Total Available Water

Anthropogenically

Available Water

Water Extraction

Water Supply

Water Demand

Water Use

Environmental Requirement

Non-extractable water

Extraction loss

Processing loss

Conveyance loss

Water Deficit
