# Supplementary material for: Sectorial Water Use Trends in the Urbanizing Pearl River Delta, China
Source: PLoS One. 2015 Feb 25;10(2):e0115039. doi: 10.1371/journal.pone.0115039 (PMC4340799; doi:10.1371/journal.pone.0115039)
Supplement: S3 Appendix — (DOCX) [file pone.0115039.s003.docx]

## Appendix S3. Chinese language references.

Research of XIONG [1] inspired development of the PRDWUM-MAN approach. In his research Xiong compared several factors affecting the industrial water use of Shaanxi Province northeast in China, and built an multi-linear regression forecast model. The affecting factors Xiong analysed in his research include industrial output (X1), ratio of heavy to light industry (X2), consumer price index (X3), water price (X4), and renovation investment (X5). Xiong’s industrial water use forecast model calculates overall industrial water use of Shaanxi province as a function of the X1, X2 and X3. We performed similar analysis based on data availability of the PRD and proposed the PRDWUM-IND as equation (4). Per capita GDP is selected instead of the industrial output is because the statistics reported industrial output include the enterprises above designate size only. It means enterprises with annual profit less than 5 million RMB are not included in the statistics during the research period. Consumer price index is neglected because it doesn’t provide much added value to model result. Web link in citation can access to the paper (in Chinese).

Reference of LI [2] discussed water use efficiency of irrigation in China. In this paper the author stated that according to the national standard, the irrigation efficiency in China should be no bigger than 0.7, 0.6 and 0.5 for irrigation district of small, medium and large scale respectively. As we analyse the overall irrigation sector of the PRD without details of spatial distribution, the medium value of 0.6 is selected. This is also included as one data source in Appendix 1.

Reference of LIU [3] is a calculation handbook for agricultural practices. In the second volume it provides several location-specific crop factors (Kc) include Guangdong province. These local values are adopted while calculating the consumptive water use of irrigated crops in the PRD following FAO approach. This is now included as one data source in Appendix 1.

Study of HE et al. [4] compared domestic water use intensity with national average, introduced several water saving policies and activities that will improve the domestic water use intensity in Guangdong province. It suggests that recently promulgated water resource management laws, regulations and raised public awareness of water saving may be the reason for disagreement between model results and WB statistics. Web link in citation can access to the paper (in Chinese).

1. Xiong Y (2005) Research on the Forecast Models for the Demand to Water in the Industry of Shaanxi Province (in Chinese). Journal of Water Resources & Water Engineering 16: http://www.cqvip.com/qk/97015a/200504/20630594.html.

2. Li Y (2003) Elementary Discussion on Water Use Efficiency of Irrigation District (in Chinese). China Rural Water and Hydropower 7.

3. Liu G (2008) Quick-speed calculation of Agriculture Handbook (Vol.2): crop cultivation (including field testing). plant protection. agricultural and water volume (Chinese Edition). Beijing, China: Chemical Industry Press.

4. He G, Kuang Y, He D, Wu S, Deng L, et al. (2006) Countermeasures for Constructing Water-Saving Society in Guangdong Province (in Chinese). Journal of China Hydrology 26: http://d.wanfangdata.com.cn/periodical_sw200605020.aspx.
