# Supplementary material for: Sectorial Water Use Trends in the Urbanizing Pearl River Delta, China
Source: PLoS One. 2015 Feb 25;10(2):e0115039. doi: 10.1371/journal.pone.0115039 (PMC4340799; doi:10.1371/journal.pone.0115039)
Supplement: S4 Appendix — (DOCX) [file pone.0115039.s004.docx]

## Appendix S4. Data Harmonization

## Harmonization of the Guangdong Water Bulletin data

### Harmonization of industrial water uses

The absolute volume of industrial water use reported by the Guangdong Water Bulletin (hereafter WB) is the overall water use by manufacturing industry and the thermal electricity industry. But, two industrial water use intensities are reported, one for overall industry, one for the manufacturers only (without the thermal electricity generation). Thus we calculated the absolute volume of manufacturing water uses by multiplying the manufacturing water use intensity with corresponding industrial value added. Water use of the thermal electricity generation was subtracted afterwards from the overall water use volume reported by the WB.

### Harmonization of domestic water use

The original water use data from WB is released as the table A(a) in billion m^3^, where DOM-Ur is the urban residential water use with urban public water use, UrbPub is the urban public water use, Resi is the residential water use in both urban and rural sectors, Resi-Ur is the urban residential water use and Resi-Ru is the rural residential water use, the latter but including also livestock water use in the data before 2003. Only two domestic water use intensity are reported however (table B(a)), i.e. urban residential water use intensity and rural residential water use intensity in litre per person-day, but consistently over time.

The data collection and reporting of the available statistics change over time. The population data used in the Water Bulletin were not consistent. For most years the “permanent population at the year-end” was used, while the “population with Hukou registration” were used in 2001 and 2002. The latter number is significantly smaller due to the large number of migrant workers in big cities like Guangzhou in China. We compute the per capita water use intensity of urban and rural sectors using the permanent population at the year-end as reported in the Guangdong Statistic Yearbook (hereafter GSY).

Thus we adjusted the original data to ensure the comparability during the research period in the following steps.

1. Collect urban and rural population from GSY. Proportion of urban population from 2001 to 2004 are not available in GSY. Data are completed by linear interpolation.
2. Volume of rural residential water use before 2008 are calculated by multiplying per capita water use intensity IRUR from WB with corresponding rural population from GSY.
3. Urban residential water use from 2003 to 2008 are extracted from overall residential water use by subtracting calculated rural residential water uses.
4. Volume of domestic water uses are harmonized into two categories as listed in Table B(b), i.e. urban domestic water use which consist urban residential and public water uses and rural domestic water use which is the rural residential water use only.

## Comparison between original WB data and harmonized data

Table A(a) Absolute Volume of the Sectorial Water Use reported by WB in 10^8^ m^3^

|  | Total | AGR | IND | DOM-Ur | UrbPub | Resi | Resi-Ur | Resi-Ru | Eco-Env |
| --- | --- | --- | --- | --- | --- | --- | --- | --- | --- |
| 2000 | 212.9 | 97.1 | 78.7 | 29.3 | - | - | - | 8.6 | - |
| 2001 | 224.7 | 96.6 | 88.8 | 30.3 | - | - | - | 9.9 | - |
| 2002 | 236.4 | 93.3 | 99.2 | 33.9 | - | - | - | 10.0 | - |
| 2003 | 249.5 | 91.2 | 107.4 | - | 16.3 | 30.6 | - | - | 4.0 |
| 2004 | 258.3 | 89.4 | 112.8 | - | 18.3 | 34.0 | - | - | 3.5 |
| 2005 | 254.7 | 86.4 | 108.5 | - | 13.2 | 39.9 | - | - | 3.5 |
| 2006 | 247.0 | 83.0 | 107.8 | - | 14.6 | 37.5 | - | - | 3.3 |
| 2007 | 249.7 | 81.0 | 104.9 | - | 14.8 | 33.6 | - | - | 4.1 |
| 2008 | 246.4 | 81.6 | 105.8 | - | 15.5 | 38.0 | - | - | 5.5 |
| 2009 | 247.5 | 80.9 | 107.0 | - | 16.2 | - | 32.5 | 5.0 | 5.8 |
| 2010 | 236.1 | 74.9 | 101.2 | - | 16.9 | - | 32.0 | 5.2 | 6.0 |

In total eight water use sectors were included, namely Agriculture (AGR), Industry (IND), Urban Domestic (DOM-Ur), Urban Public (UrbPub), Residential (Resi), Urban Residential (Resi-Ur), Rural Residential (Resi-Ru) and Eco-Environmental compensation (Eco-Env)

Table A(b) Harmonized Sectorial Water Use in 10^8^ m3.

|  | Total | AGR | MAN | ELE | DOMU | DOMR |
| --- | --- | --- | --- | --- | --- | --- |
| 2000 | 212.9 | 97.1 | - | - | 29.2 | - |
| 2001 | 224.7 | 96.6 | 58.8 | 30.0 | 31.5 | 6.9 |
| 2002 | 236.4 | 93.3 | - | - | 34.7 | 7.5 |
| 2003 | 249.5 | 91.2 | - | - | 40.5 | 6.4 |
| 2004 | 258.3 | 89.4 | 85.0 | 27.8 | 46.7 | 5.6 |
| 2005 | 254.7 | 86.4 | 78.9 | 29.6 | 46.5 | 4.9 |
| 2006 | 247.0 | 83.0 | 79.5 | 28.3 | 51.8 | 5.1 |
| 2007 | 249.7 | 81.0 | 78.1 | 26.8 | 50.2 | 4.9 |
| 2008 | 246.4 | 81.6 | 73.3 | 32.5 | 48.7 | 4.9 |
| 2009 | 247.5 | 80.9 | 68.8 | 38.2 | 48.8 | 5.0 |
| 2010 | 236.1 | 74.9 | 70.6 | 30.6 | 48.9 | 5.2 |

Agriculture (AGR), Manufacturing Industry (MAN), Thermal Electricity Industry (ELE), Urban Domestic (DOMU), and Rural Domestic (DOMR)

Table B(a) Reported Sectorial Water Use Intensity

|  | I_Total_ | I_AGR_ | I_MAN_ | I_IND_ | I_UR_ | I_RU_ |
| --- | --- | --- | --- | --- | --- | --- |
|  | (m^3^/person) | (m^3^/ha) | (m^3^/10^4^VA) | (m^3^/10^4^VA) | (l/day) | (l/day) |
| 2000 | 513 |  | - | - | 283 | 201 |
| 2001 | 988 | 11475 | 188 | 264 | 290 | 155 |
| 2002 | 981 | 11415 | - | - | 295 | 178 |
| 2003 | - | 10620 | - | - | 198 | 158 |
| 2004 | 653 | 12840 | 127 | 182 | 250 | 168 |
| 2005 | 554 | 12225 | 96 | 140 | 248 | 155 |
| 2006 | 560 | 11235 | 78 | 112 | 260 | 160 |
| 2007 | 556 | 12120 | 65 | 98 | 252 | 136 |
| 2008 | 540 | 11895 | 49 | 78 | 239 | 145 |
| 2009 | 534 | 11835 | 45 | 78 | 235 | 161 |
| 2010 | 441 | 9210 | 37 | 66 | 201 | 148 |

Table B(b) Harmonized Sectorial Water Use Intensity

|  | I_Total_ | I_IND_ | I_DOMU_ | I_DOMR_ | I_DOM-Total_ |
| --- | --- | --- | --- | --- | --- |
|  | (m^3^/person) | (m^3^/10^4^VA) | (l/day) | (l/day) | (l/day) |
| 2000 | 496 | 289 | 269 | - | - |
| 2001 | 513 | 284 | 278 | 150 | 241 |
| 2002 | 535 | 264 | 296 | 169 | 261 |
| 2003 | 559 | 222 | 335 | 152 | 288 |
| 2004 | 572 | 168 | 374 | 139 | 317 |
| 2005 | 560 | 132 | 362 | 129 | 309 |
| 2006 | 521 | 106 | 377 | 146 | 329 |
| 2007 | 506 | 87 | 349 | 136 | 306 |
| 2008 | 480 | 71 | 323 | 133 | 286 |
| 2009 | 462 | 70 | 308 | 134 | 275 |
| 2010 | 420 | 53 | 288 | 148 | 264 |
