# Supplementary material for: Sectorial Water Use Trends in the Urbanizing Pearl River Delta, China
Source: PLoS One. 2015 Feb 25;10(2):e0115039. doi: 10.1371/journal.pone.0115039 (PMC4340799; doi:10.1371/journal.pone.0115039)
Supplement: S5 Appendix — (DOCX) [file pone.0115039.s005.docx]

## Appendix S5. Top 10 Manufacturers in the PRD

| Rank | Manufactures | PRD | Guangzhou | Shenzhen | Zhuhai | Dongguan | Zhongshan | Foshan | Huizhou | Jiangmen | Zhaoqing |
| --- | --- | --- | --- | --- | --- | --- | --- | --- | --- | --- | --- |
| 1 | Telecommunications Equipment, Computers and Other Electronic Equipment | 22% | 10% | 47% | 20% | 25% | 12% | 5% | 43% | 5% | 11% |
| 2 | Electric Machinery and Equipment | 12% | 6% | 7% | 25% | 10% | 20% | 23% | 7% | 10% |  |
| 3 | Production and Distribution of Electric Power and Heat Power | 7% | 6% | 7% | 10% | 13% | 3% | 5% | 4% | 6% | 6% |
| 4 | Transport Equipment | 6% | 21% |  | 2% |  |  |  | 3% | 10% |  |
| 5 | Raw Chemical Material and Chemical Products | 6% | 11% |  | 5% |  | 4% | 3% | 10% | 6% | 6% |
| 6 | Metal Products | 4% | 3% | 3% |  |  | 7% | 8% | 3% | 14% | 10% |
| 7 | Plastic | 4% |  | 3% | 4% | 5% | 6% | 5% | 3% |  |  |
| 8 | Non-metallic Mineral Products | 3% |  |  |  |  |  | 9% |  | 4% | 6% |
| 9 | Textile Garments, Footwear and Caps | 3% | 3% | 1% | 2% | 4% | 7% | 3% |  | 5% | 4% |
| 10 | Textile Industry | 3% |  |  |  | 4% | 5% | 4% | 2% | 6% | 6% |
| 11 | Petroleum and Natural Gas Extraction | 3% |  | 9% |  |  |  |  |  |  |  |
| 12 | Measuring Instrument and Machinery for Cultural Activity and Office Work | 2% |  | 3% | 4% | 3% |  |  |  |  |  |
| 13 | Leather, Fur, Feather and Related Products | 2% |  |  |  | 4% | 4% |  | 3% |  | 6% |
| 14 | General Purpose Equipment | 2% | 3% |  |  |  |  |  |  |  |  |
| 15 | Paper and Paper Products | 2% |  |  |  | 5% | 3% |  |  |  |  |
| 16 | Special Purpose Equipment | 2% |  | 2% |  |  |  |  |  |  |  |
| 17 | Manufacturing and Processing of Non-ferrous Metals | 2% |  |  |  |  |  | 6% |  |  | 6% |
| 18 | Food Manufacturing | 2% |  |  |  |  |  |  |  | 4% |  |
| 19 | Cultural, Educational and Sport Goods | 1% |  |  |  | 3% |  |  |  |  |  |
| 20 | Handicrafts and Others | 1% |  | 2% |  |  |  |  |  |  |  |
| 21 | Medical and Pharmaceutical Products | 1% |  |  | 6% |  |  |  |  |  |  |
| 25 | Manufacturing and Processing of Ferrous Metals | 1% | 4% |  | 2% |  |  |  |  |  |  |
| 27 | Processing of Petroleum, Coking, Processing of Nucleus Fuel | 1% | 5% |  |  |  |  |  | 7% |  |  |
| 31 | Processing of Timbers, Wood, Bamboo, Cane, Palm Fiber and Straw Products | 0% |  |  |  |  |  |  |  |  | 4% |

Proportion of the manufacture in the overall industrial value-added
